# La trichotillomanie des cils: à propos d’un cas

**DOI:** 10.11604/pamj.2017.28.142.13822

**Published:** 2017-10-14

**Authors:** Khadija Drissi Touzani, Zineb Lamari, Fouad Chraibi, Meryem Abdellaoui, Idriss Benatiya Andaloussi

**Affiliations:** 1 Service d’Ophtalmologie, CHU Hassan II, Fès, Maroc

**Keywords:** Trichotillomanie, cils, thérapie cognitive et comportementale, Trichotillomania, eyelashes, cognitive-behavioral therapy

## Abstract

La trichotillomanie est un trouble psychiatrique négligé qui pousse à s'arracher les cheveux, mais aussi les sourcils ou tout autre poil dont rarement les cils. Elle est définie dans le Manuel diagnostique et Statistique des Troubles Mentaux en tant que trouble des habitudes et des impulsions. A travers cette observation, nous rapportons le cas d'une fille qui souffre de trichotillomanie des cils de l'œil gauche suite à une faible estime de soi. La maladie peut être épisodique mais généralement chronique et difficile à traiter. Sur la base des éventuelles complications médicales et psychiatriques, il est important que le diagnostic soit exact et précoce. Le traitement de référence repose sur la thérapie cognitive et comportementale.

## Abstract (en)

Trichotillomania is a neglected psychiatric disorder characterized by the urge to pull out the hair, the eyebrows or any other hair, but rarely the eyelashes. In the Diagnostic and Statistical Manual of Mental Disorders it is defined as habits and pulses disturbance. We here report the case of a girl with trichotillomania involving her left eyelashes due to low self-esteem. The disease may be episodic but it is usually chronic and difficult to treat. Given the possible clinical and psychiatric complications, early and accurate diagnosis is essential. Cognitive-behavioral therapy is the gold standard treatment.

## Introduction

La trichotillomanie des cils, rarement rapportée dans la littérature est caractérisé par l’arrachage compulsif de ses propres cils. Nous rapportons le cas d’une jeune fille ayant ce tic.

## Patient et observation

**Figure 1:**
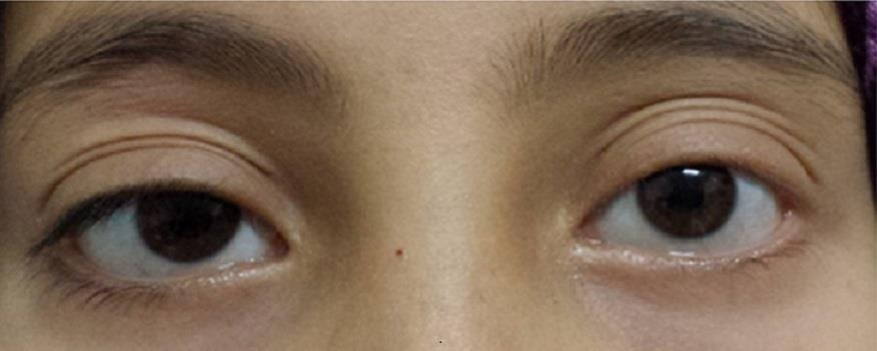
Fille en vue de face: noter la raréfaction des cils au niveau de l’œil gauche

**Figure 2:**
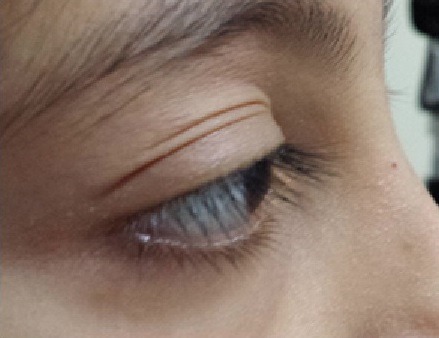
Aspect normal des bords libres de l’œil droit en vue de profil

**Figure 3:**
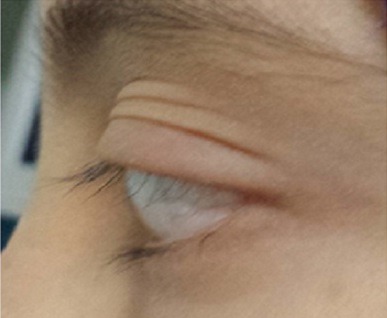
Cils raréfiés au niveau des bords libres de l’œil gauche en vue de profil

Une fille de 12 ans consulte avec son père pour chute spontanée des cils de l’œil gauche. L’interrogatoire ne note pas d’antécédents particulier notamment pas de tares connues, pas d’antécédents ophtalmologiques, pas d’antécédents dermatologiques. L’histoire de la maladie remonte à une année où la famille a constaté une raréfaction unilatérale des cils de l’œil gauche (Figure 1). L’examen ophtalmologique trouve au niveau de l’œil droit une acuité visuelle à 12/10^ème^, l’examen des annexes notamment les bords libres (Figure 2) est normal, le segment antérieur et postérieur sont sans particularités. Au niveau de l’œil gauche: acuité visuelle à 12/10^ème^, l’examen des annexes trouve bonne statique et dynamique palpébrale, bord libre d’aspect normale, cils bien implantés mais très raréfiés (Figure 3), peau palpébrale d’aspect normale sans croûte ni sécrétions, pas d’entropion ni d’ectropion, pas de blépharite, le segment antérieur et postérieur sont normaux. Tonus oculaire à 12 mmhg au niveau des 2 yeux. L’examen dermatologique est normale notamment pas d’alopécie. Le reste de l’examen somatique est sans particularités. Un bilan initial a été demandé à la recherche d’une anémie ou d’un trouble hormonal est revenu normal. Devant la normalité du bilan ainsi que l’unilatéralité du symptôme, on a procédé à une reprise d’un interrogatoire approfondi avec la fille toute seule (Fille timide et son père qui parle toujours). La jeune avoue enfin qu’elle arrache ses cils et ceci depuis son échec à l’examen final de la 6^ème^année primaire, sa famille et ses amies se moquent d’elle. Selon la fille, c’est le seul moyen de se soulager et de déstresser. Le diagnostic de Trichotillomanie des cils a été retenu. On a convaincu le père de la nécessité de voir un psychiatre. La fille a consulté un psychiatre pendant quelques mois, mais elle n’a pas pu vaincre cette automutilation.

## Discussion

La trichotillomanie est un trouble du comportement qui pousse à s'arracher les cheveux, mais aussi les sourcils, les cils ou tout autre poil [1]. Cette maladie fait partie des troubles obsessionnels compulsifs (TOC). Elle se manifeste le plus souvent chez l'enfant et l'adolescent, particulièrement en période de stress. Il s'agit alors d'un simple tic, mais cette maladie d'origine psychologique peut perdurer à l'âge adulte. Selon les études, on estime qu’entre 1 et 2% de la population souffre de trichotillomanie [2]. 17 à 75% des trichotillomanes cachent leur trouble à leur entourage [3]. Souvent la trichotillomanie apparaît après un stress mais elle peut également commencer sans raison (arrachage automatique). La trichotillomanie est fortement associée à une image négative de soi et particulièrement de son corps, une anxiété et une frustration importante, de la dépression, une faible estime de soi. Les études n'ont pas encore montré de cause certaine. Des chercheurs ont identifié une mutation sur un gène chez des sujets atteints de trichotillomanie [4]. Le traitement de référence est la thérapie cognitive et comportementale (TTC) [5]. Celle-ci consiste via une approche toujours personnalisée à modifier progressivement les comportements et les réflexes. Malgré tout, ce trouble reste difficile à traiter. C’est pourquoi dans certains cas, des médicaments psychotropes (antidépresseurs, anxiolytiques, neuroleptiques…) sont encore prescrits mais en complément de la TTC.

## Conclusion

Mieux comprendre cette maladie et connaître ses origines représentent des étapes importantes pour améliorer l’efficacité du traitement.

## Conflits d’intérêts

Les auteurs ne déclarent aucun conflit d'intérêt.
